# Plerixafor Salvage Is Safe and Effective in Hard-to-Mobilize Patients Undergoing Chemotherapy and Filgrastim-Based Peripheral Blood Progenitor Cell Mobilization

**DOI:** 10.1155/2012/931071

**Published:** 2012-04-10

**Authors:** Farrukh T. Awan, S. Thomas Kochuparambil, David DeRemer, Aaron Cumpston, Michael Craig, Anand Jillella, Mehdi Hamadani

**Affiliations:** ^1^Bone Marrow Transplant Program, Section of Hematology/Oncology, Department of Medicine, Georgia Health Sciences University, Augusta, GA 30912, USA; ^2^Department of Pharmacy, Georgia Health Sciences University, Augusta, GA, USA; ^3^Osborn Hematopoietic Malignancy and Transplant Program, West Virginia University, Morgantown, WV 26506, USA

## Abstract

The combination of filgrastim (G-CSF) and plerixafor is currently approved for mobilizing peripheral blood progenitor cells in patients with non-Hodgkin lymphoma and multiple myeloma undergoing autologous peripheral blood hematopoietic cell transplantation. However, chemotherapy and G-CSF-based mobilization remains a widely used strategy for peripheral blood progenitor cell collection. In this paper we describe our experience from two North American transplant centers in a series of patients who received salvage plerixafor while failing chemotherapy and G-CSF mobilization. Patients received a median of two doses of plerixafor salvage upon failure to mobilize adequate number of peripheral blood progenitor cells at neutrophil recovery. The use of plerixafor was associated with a 2.4-fold increase in peripheral blood CD34+ cell count and 3.9-fold increase in total CD34+ cell yield. All patients were able to collect ≥2 × 10^6^ CD34+ cells/kg with this approach. These results were more pronounced in patients with a higher CD34+ cell count at the time of the first plerixafor dose. Interestingly, peripheral blood white blood cell count was not shown to correlate with a response to plerixafor. Our results provide safety and efficacy data for the use of plerixafor in patients who are destined to fail chemomobilization.

## 1. Introduction

Autologous peripheral blood hematopoietic cell transplantation (auto-PBHCT) is a well-established therapeutic option for patients with a variety of hematologic malignancies. Mobilization of peripheral blood progenitor cells (PBPC) for auto-PBHCT can be accomplished by using cytokines, most commonly granulocyte-colony stimulating factor (G-CSF), either alone or in combination with chemotherapy (e.g., cyclophosphamide, etoposide, cytarabine, etc.) or plerixafor [1, 2]. Recently reported phase III studies have also shown superiority of the combination of G-CSF with plerixafor over G-CSF alone for mobilizing PBPC in patients with non-Hodgkin lymphoma (NHL) and multiple myeloma (MM) [3, 4].

Plerixafor acts by selective and reversible antagonism of CXCR4 on CD34+ hematopoietic stem cells (HSC). This results in disruption of its interaction with CXCL12 (formally SDF1) on bone marrow stromal cells, that cause a rapid release of stem and progenitor cells from bone marrow into peripheral blood. While plerixafor-based PBPC mobilization can circumvent the need for chemotherapy to mobilize CD34+ PBPCs, to our knowledge no prospective trials comparing plerixafor plus G-CSF to chemomobilization have been published to date. Limited data on murine models suggest that a combination of plerixafor and chemotherapy may be more effective than the use of plerixafor alone for PBPC mobilization [5].

Despite the promising results of plerixafor and G-CSF for PBPC mobilization in patients with MM and NHL [3, 4, 6–8], the use of chemotherapy and G-CSF-based regimens to mobilize PBPC remains standard practice in many transplant centers. This decision is often influenced by a desire to improve collection yield, reduce mobilization failures especially in patients who are elderly, heavily pre-treated, and have poor bone marrow cellularity, and/or as an attempt to provide disease control [9–11]. Limited data are available on the preemptive use of plerixafor *salvage* in patients failing to collect adequate numbers of PBPC with chemotherapy and G-CSF-based mobilization [12–14], and this topic has been reviewed recently [15]. Herein we report our experience from two North American transplant centers in a series of patients who received plerixafor salvage while failing chemotherapy and G-CSF mobilization.

## 2. Methods

For patients undergoing chemotherapy and G-CSF-based mobilization, it is standard operating procedure at both transplant centers to measure peripheral blood CD34+ cell count daily when the patient's white blood cell (WBC) count recovers to ≥4,000/*μ*L or from day +12 (after chemotherapy) onwards (whichever occurs first). Apheresis is initiated when the peripheral blood CD34+ cell count is ≥10/*μ*L. Patients *destined* to fail PBPC chemomobilization were defined as (i) those with a peak peripheral blood CD34+ cell count of <10/*μ*L following WBC count recovery (WBC count of ≥4,000/*μ*L) after chemotherapy-induced nadir or (ii) those who failed to collect at least ≥1 × 10^6^ CD34+ cells/kg after two apheresis sessions. In these patients failing chemomobilization, we administrated plerixafor at a dose of 0.24 mg/kg subcutaneously 10 hours prior to apheresis in conjunction with G-CSF (10 *μ*g/kg), as a preemptive salvage strategy. All collections were performed with a COBE Spectra Apheresis System (CaridianBCT, Lakewood, CO), by processing three to four blood volumes. It is the institutional policy at both transplant centers to routinely target collection of 5 × 10^6^ CD34+ cells/kg. Determination of peripheral blood CD34+ cell count and CD34+ cell content of the apheresis product was performed at the Georgia Health Sciences University HLA Laboratory and West Virginia University Hospitals Flow Cytometry Laboratory. The BD FACSCanto II flow cytometer, (Becton Dickinson, San Jose, CA) was used for all analyses. After red blood cell lysis, washed samples were used for CD34+ enumeration with PE-labeled, 8G12 clone, immunoglobulin G1 (Becton Dickinson, San Jose, CA) based on International Society of Hematotherapy and Graft Engineering (ISHAGE) guidelines. The final products were cryopreserved in 10% DMSO using a controlled rate freezer and stored in liquid nitrogen. Successful mobilization was defined as a total of ≥2 × 10^6^ CD34+ cells/kg patients body weight in the final product. Data was collected on mobilization and transplant outcomes through an electronic data base, prospectively maintained at each participating institution and analyzed utilizing SPSS version 13.0.

## 3. Results

Patient characteristics and transplantation outcomes of 16 patients who were failing chemomobilization (as defined above) and received preemptive plerixafor are summarized in Table 1. The median age was fifty-six years. Patients had received a median of two lines of therapies (range 1–3) prior to PBPC mobilization. After recovering from chemotherapy-induced count nadir (i.e., WBC ≥ 4000/*μ*L), 15 patients had a peak peripheral blood CD34+ cell count of <10 *μ*L. Five patients underwent at least 2 sessions of apheresis but were unable to collect ≥1 million CD34+ cells/kg. These patients subsequently received a median of two doses of plerixafor salvage (range 1–8). The median number of apheresis sessions was 3.5 (range 2–7), and the median number of CD34+ cells collected was 3.9 × 10^6^ cells/kg (range 2.4–7.8). Utilizing a cutoff of ≥2 × 10^6^ CD34+ cells/kg, all patients who received plerixafor had a successful collection. Nineteen percent of the patients were able to collect ≥5 × 10^6^ CD34+ cells/kg. Three patients (one with Hodgkin lymphoma and two with NHL) required more than four doses of plerixafor, but all eventually collected ≥2 × 10^6^ CD34+ cells/kg. The median peak peripheral blood CD34+ cell count prior to plerixafor administration was 3.5/*μ*L (range 0–15) and increased to 6/*μ*L (range 2–47) after the first dose of plerixafor (*P* = 0.03). 93% of the patients had a peak peripheral blood CD34+ cell count of <10/*μ*L before plerixafor salvage. Four patients had a peak peripheral blood CD34+ cell count of ≤1/*μ*L before plerixafor salvage. Kinetics of peripheral blood CD34+ cell and WBC count changes after each dose of plerixafor for these 4 patients is shown in Table 2. After transplantation, the median time to neutrophil and platelet engraftment was 10 days (range 9–15) and 20 days (range 9–29), respectively.

In order to identify predictors of response to plerixafor salvage, correlation analyses were performed on a variety of factors. As expected, patients with a higher peripheral blood CD34+ cell count at the time of the first plerixafor dose had a higher magnitude of change in their peripheral blood CD34+ cell counts (*r* ^2^ = 0.58, *P* = 0.01). Only three patients had a CD34+ cell count of ≥10/*μ*L before the first dose of plerixafor, and their median increase was 18/*μ*L compared to 6/*μ*L for patients who had peripheral blood CD34+ cell counts of <10/*μ*L however, this difference was not statistically significant (*P* = 0.3). We did observe a positive correlation between peak peripheral blood CD34+ cell count before the first dose of plerixafor and the total number of CD34+ cells collected at apheresis (*r* ^2^ = 0.62; *P* = 0.01). Of the 41 collections with plerixafor, the mean CD34+ cell dose collected was 0.79 × 10^6^ CD34+ cells/kg from 25 collections in patients with a peripheral blood CD34+ cell count <10/*μ*L versus 2.09 × 10^6^ CD34+ cells/kg from 16 collections in patients with a CD34+ cell count greater than 10/*μ*L (*P* = 0.001). Correlation analyses were performed in order to define an optimal cutoff of WBC count that can be used as a marker for the initiation of plerixafor salvage, which showed that WBC count had no correlation with a change in CD34+ cell count after the first dose of plerixafor (*r* ^2^ = −0.21, *P* = 0.41). Utilizing the median WBC count of 32/*μ*L at the time of administration of plerixafor in our patients, we found that we were able to collect a higher number of CD34+ cells in patients who had a WBC count ≤32/*μ*L as compared to those with a WBC count >32/*μ*L (1.67 × 10^6^/kg versus 0.8 × 10^6^/kg, *P* = 0.02 resp.).

## 4. Discussion

Our limited multicenter outcomes data suggest that the addition of plerixafor as a preemptive salvage may rescue patients who are destined to fail chemotherapy and G-CSF-based PBPC mobilization. In our series we used plerixafor salvage to rescue an otherwise failed attempt for chemomobilization, which contrasts with prior studies where plerixafor was used to *remobilize* patients who had failed prior mobilization attempts [16]. This is also in contrast to studies where plerixafor was routinely given to patients undergoing chemomobilization [17].

In our series plerixafor was given after recovery from chemotherapy-induced count nadir (median of 11.5 days after chemotherapy) and resulted in successful CD34+ cell collection in all patients, who were otherwise likely to fail chemomobilization. Interestingly patients with a WBC count of ≤ 32/*μ*L were able to collect a higher number of CD34+ cells. This is in contrast to earlier data [12–15] that indicated limited efficacy of plerixafor in patients with a lower WBC count. This discrepancy can be a reflection of the decreased efficiency of the collection process in patients with a higher WBC count or possibly a reflection of timing of plerixafor administration. Generally a CD34+ cell count of 10–13/*μ*L is used as a cutoff for initiating apheresis following cytokine only, cytokine plus plerixafor, or chemomobilization in majority of transplant centers in the country. The median CD34+ cell count of our patients was only 3.5/*μ*L with 82% less than 10/*μ*L at the time of the first plerixafor dose, and all patients were able to collect a minimum of 2 × 10^6^ cells/kg. However, a valid cutoff for peripheral blood CD34+ cell count to initiate apheresis when plerixafor is used as a salvage for failed chemomobilization is unknown.

Patient characteristics and institutional preference will likely continue to influence the choice for mobilization strategy in patients undergoing an auto-PBHCT [7]. While no prospective data are available to demonstrate better efficacy or cost effectiveness of plerixafor-based mobilization over chemotherapy-based mobilization [18], our preliminary data provide safety and efficacy data for plerixafor salvage to rescue patients failing chemotherapy-based PBPC mobilization.

## Figures and Tables

**Table 1:** Baseline characteristics and mobilization outcomes (*N* = 16).

Median age in years (range)	56 (20–71)
Gender	56% male
Race	63% Caucasian
	37% African American
Disease subtypes	37% non-Hodgkin lymphoma
	44% multiple myeloma
	19% others^1^
Mobilization chemotherapy regimens	69% cyclophosphamide^2^
	31% others^3^
Disease status at the time of transplant	37% complete remission
	37% partial remission
	13% stable disease
	13% progressive disease
Median number of prior therapies (range)	2 (1–3)
Prior radiation therapy	19%
Median number of days of G-CSF administration after chemotherapy prior to the administration of plerixafor (range)	12 (7–21)
Median number of days to plerixafor administration after chemotherapy (range)	16 (12–25)
Mean white blood cell count (/*μ*L) at the time of starting plerixafor Therapy (range)	13.4 (4.1–25.2)
Mean absolute neutrophil count (/*μ*L) at the time of starting plerixafor Therapy (range)	11.7 (3.5–20.7)
Median peripheral blood CD34+ cell count (/*μ*L) at the time of starting plerixafor therapy (range)	3.5 (0–15)
Median peripheral blood CD34+ cell count (/*μ*L) at the time of starting apheresis (range)	8.5 (2–30)
Median number of plerixafor doses (range)	2 (1–8)
Median number of apheresis sessions (range)	3.5 (2–7)
Median number of CD34+ cells (×10^6^ cells/kg) collected prior to starting plerixafor (range)	0 (0–3.13)
Median number of CD34+ cells (×10^6^ cells/kg)collected	3.9 (2.4–7.8)
Percentage of patients with >2 × 10^6^ cells/kg CD34+ cells collected	100%
Percentage of patients with >5 × 10^6^ cells/kg CD34+ cells collected	19% (3/16)
Median increase in CD34+ cell count (/*μ*L) after first dose of plerixafor (range)	6.5 (1–35)

^1^Other diagnoses included Hodgkin lymphoma = 2 and Ewing sarcoma = 1.

^2^Cyclophosphamide dose = 3-4 gm/m^2^ intravenously.

^3^Other chemotherapies included ICE (ifosfamide, carboplatin and etoposide), Hyper-CVAD (cyclophosphamide, vincristine, doxorubicin and dexamethasone), and D-PACE (dexamethasone, cisplatin, doxorubicin, cyclophosphamide, and etoposide).

**Table 2:** Peripheral blood CD34+ cell and WBC count kinetics in response to plerixafor salvage in patients with extremely low baseline CD34+ cell counts.

	PB counts (/*μ*L)	Baseline	Post P dose no. 1	Post P dose no. 2	Post P dose no. 3	Post P dose no. 4	Post P dose no. 5	Post P dose no. 6	Post P dose no. 7	Post P dose no. 8
Patient #1	WBC	28.5	25.8	24.7	27.3	43.3				
	CD34+	1	1	1	2	4				

Patient #2	WBC	14	22.6	26.2	28.9	31.3	34.1	38.4	46.1	
	CD34+	1	2	2	4	4	6	7	11	

Patient #3	WBC	4.1	10.6	14.7	14.8	17.1	18.6	20	22.7	27
	CD34+	0	3	4	3	3	8	9	6	12

Patient #4	WBC	19.4	51.6	60.8	58.8	58.1	66.9	80.1		
	CD34+	0	3	2	4	4	13	13		

P = plerixafor; PB = peripheral blood; WBC = white blood cell count.
